# Integrating density and fertilizer management to optimize the accumulation, remobilization, and distribution of biomass and nutrients in summer maize

**DOI:** 10.1038/s41598-020-68730-8

**Published:** 2020-07-16

**Authors:** Hao Ren, Yi Cheng, Rongfa Li, Qinglong Yang, Peng Liu, Shuting Dong, Jiwang Zhang, Bin Zhao

**Affiliations:** State Key Laboratory of Crop Biology and College of Agronomy, Shandong Agricultural University, Tai’an , 271018 Shandong China

**Keywords:** Plant sciences, Plant ecology, Plant physiology

## Abstract

Improved the utilization of fertilizer while maintaining the increased of grain yield was the focus of Chinese researchers. Nutrient uptake, distribution, and remobilization are important factors affecting the fertilizer utilization and grain yield of maize. This study aimed to provide a theoretical and practical basis for science-based, high-yielding, and high-efficiency cultivation practices by examining differences in biomass and nutrient uptake, distribution, and remobilization characteristics under three cultivation patterns. We set 12 treatments as follows: super high-yielding cultivation pattern (SH), optimized nutrient management cultivation pattern (ONM), local farmer's practice cultivation pattern (FP), and a series of nutrient omission plots, which excluded nitrogen (N), phosphorus (P), or potassium (K) from the three patterns. The results demonstrated that SH and ONM increased the yield and actual harvested ears by 35.4, 20.7 and by 20.2, 17.6%, respectively. Compared with the FP, SH and ONM increased biomass, N, P, and K accumulation at silking (R1 stage) by 24.4, 31.2, 39.4, and 34.8%, and by 21.7, 22.2, 31.7, and 34.8%, respectively. SH and ONM significantly increased biomass and nutrient distribution to the grains. ONM significantly increased N use efficiency. P and K use efficiency under the ONM pattern was significantly higher than under SH, but was lower than under the FP pattern over two years. This research demonstrates that ONM may significantly reduce fertilizer rates, effectively improve the nutrient remobilization efficiency and uptake at post-silking without negatively affecting grain yield, thereby increasing N use efficiency.

## Introduction

With the replacement of varieties and the development of cultivation techniques, average global maize yield has increased significantly^1–3^. Maize became China’s largest food crop in 2012^4^. Yet, the high grain yield of maize in China depends on a large amount of fertilizer inputs, which cause fertilizer losses and waste resources. Meanwhile, N fertilizer is often applied at rates exceeding plant requirements, thus inducing lower agronomic N use efficiency (N AE, kg grain yield increase per kg N applied) of maize in China than the average N AE worldwide. This has unintended environmental consequences such as nitrate leaching and the emission of nitrous oxide and ammonia^5–7^. Therefore, it is urgent to improve the efficiency of fertilizer utilization while avoiding pollution and achieving high yields.

Plant nutrients, including nitrogen, phosphorus, and potassium, play important roles in the growth and development of maize^8,9^. The nutrient accumulation in maize kernels is derived from the nutrient absorption during the reproductive growth period and the nutrient redistribution during the vegetative growth period^9,10^. 25–82% of grain N^11,12^, 1–75% grain P and 45–447% grain K contributions from contributed by vegetative nutrients accumulate during pre-silking in maize^13^. However, there is a strong negative correlation between nitrogen remobilization and absorption during reproductive growth period^3,14,15^. Significantly, Photosynthetic activity in leaves is maintained on the basis of higher nitrogen concentrations in the leaves during the grain-filling period^16^, but this physiological requirement may hinder the migration of nitrogen from vegetative organs to reproductive organs^17^.Therefore, to achieve high yields and high efficiency we must coordinate post-silking N absorption and remobilization.

Balance the nutrients remobilization from vegetative organs to reproductive organs and the absorption of nitrogen at post-silking is the key to improve nutrient utilization efficiency^18,19^. These growth processes are influenced by maize varieties and cultivation practices^10,18,20^. In modern breeding, stay-green varieties have a higher photosynthetic duration, dry matter accumulation and nutrient absorption in the late growth period^21^, but they do leave more nutrients in the vegetative organs^22^. Studies have shown that low-N stress can lead to the decrease of mineral nutrient concentration in grains but promote the remobilization of nutrients in vegetative organs^12^. Under low density condition, N application could promote the accumulation of N in maize, but as the amount of nitrogen increases, the N remobilization efficiency, the remobilization amount, and the distribution to the grain may decrease^23^. Increasing the planting density can effectively improve the total N accumulation and N remobilization of maize, so as to obtain high nutrient remobilization efficiency^24,25^. When plant density had a positive effect on N recovery efficiency and N internal efficiency of maize plants, higher planting density had an effect on nitrogen utilization efficiency of medium and high nitrogen application^26^. The application of nitrogen fertilizer smoothed the resource competition between plants and the variation between plants and ear under the condition of high density. However, this effect was more pronounced in maize crops with density-tolerant varieties than with density-intolerant varieties^27^. It has also been suggested that the trait of stay green maybe a substitute for increasing nitrogen accumulation after silking, as this causes nitrogen to be unable to be remobilization from vegetative part of the plant, and that increasing density can regulate this trait and keep maize yields up^28^. The stay green of maize leaves and plant density affect the accumulation and remobilization of nitrogen after silking^29^. Therefore, planting stay green cultivars, increasing density and reducing nitrogen have become the mainstream measures to improve yield and nitrogen utilization efficiency^30^. Field studies showed that the absolute value of nutrient absorption and dry matter accumulation was high in the late stage of silking, and there was a strong correlation between the grain yield and its component factors^31^. According to the nutrient absorption characteristics of maize in different periods, changing the fertilization method, especially the fertilization method, could improve maize yield and nitrogen absorption^32^. Researchers have studied the effects of varieties and single management practices (density and fertilizer) on maize N accumulation and N remobilization^12^, and found that the combination of varieties and agronomic management measures could increase grain yield and grain N concentration while reducing N input^33^, but there are few studies on the plant remobilization characteristics of P and K, and these studies often ignore the coordination between post-silking nutrient uptake and nutrient remobilization.

Optimizing cultivation practices is an important approach used to obtain high maize yield and high nutrient efficiency^1^. However, few studies have investigated the effects of different cultivation patterns on the nutrient remobilization characteristics of maize to improve nutrient use efficiency. We hypothesized that appropriate cultivation management could simultaneously improve food yield and nutrient utilization efficiency by balancing nutrient production and nutrient remobilization. We set up three different cultivation patterns: SH, ONM and FP, whose fertilizer management and density control are integrated. The concept of SH treatment design is based on the maximum yield available under the condition of unlimited nutrients. Therefore, its input is no matter the cost, the maximum amount of fertilizer applied, the maximum number of fertilization and the highest planting density are adopted. The idea of ONM treatment design is to optimize partial fertilizer application amount, fertilization frequency and planting density based on SH, so as to obtain higher fertilizer utilization efficiency and keep the yield unchanged or slightly decreased. FP imitates the planting mode of local farmers, so the planting density and fertilizer application rate are low. Our study investigated the accumulation, distribution, and remobilization of biomass, N, P, and K in different vegetative organs (leaves, stalks, and husk and cob) at post-silking, and compared their differences under three cultivation patterns with stay green variety “ZD958” in successive years. The purpose of our study was to understand the remobilization characteristics of N, P, and K during grain filling stage in relation to nutrient use efficiency under different cultivation patterns.

## Materials and methods

### Sites and experimental conditions

The experiments were conducted in 2014 and 2015 at the Wenkou Experimental Station, Shandong Agricultural University, Taian. The Wenkou station is located at 35°58′10 N, 117°03′30 E and 178 m above sea level. The soil type is a typical brown soil with a sandy loam texture. The chemical characteristics of the 0–40 cm soil layer in 2014 and 2015 are shown in Table 1.

**Table 1:** Basic chemical properties of the soil in the tillage layer (0–40 cm).

Site	Year	Soil layers (cm)	Organic matter (g kg^−1^)	Total N (g kg^−1^)	Alkali-hydrolyzable N (mg kg^−1^)	Available P (mg kg^−1^)	Available K (mg kg^−1^)
Wenkou	2014	0–20	15.16	1.29	98.46	37.14	120.68
		20–40	14.28	1.35	110.42	35.12	128.41
	2015	0–20	16.18	1.91	120.24	48.12	108.16
		20–40	15.55	1.99	130.42	44.38	111.26

### Treatments and experimental design

The maize stay green variety “ZD958”, which is commonly planted in northern China, was used as the experimental variety. All on-farm experiments followed a standardized experimental protocol. We set up 12 treatments for the experiment, as follow: super high-yielding cultivation pattern (SH), optimized nutrient management cultivation pattern (ONM), local farmer's practice cultivation pattern (FP), and a series of nutrient omission plots, which excluded N, P, or K from the three cultivation patterns (Only used to calculate nutrient utilization efficiency). The replications were fully randomized in a 12 × 4 randomized block design. Each plot was 180 m^2^. Plots were separated by 30-cm wide earthen dams to allow flood irrigation of each plot. The experimental blocks were separated by 1-m walkways. N, P, and K were provided as urea (containing 46% N), superphosphate (containing 12% P_2_O_5_), and potassium chloride (containing 60% K_2_O), respectively. We used chicken manure as organic fertilizer (which contained 315 g kg^–1^ organic carbon (dry basis), 32.3 g kg^–1^ P_2_O_5_, and 30.4 g kg^–1^ K_2_O)^34^.

In the SH, plant density was 90,000 plants ha^–1^ (Maximum planting density allowed by local climatic conditions) and fertilization rates were as follows: 450 kg N ha^−1^, 270 kg P ha^−1^, 450 kg K ha^−1^, and 15,000 kg organic fertilizer ha^−1^. All of the P and chicken manure was applied as a base fertilizer for each treatment. N was split into two forms for application: a base fertilizer and one dressed at the following growth stages: 6-leaf stage (V6), 12-leaf stage (V12), anthesis (VT), and one week after the VT stage with a ratio of 1:2:3:2:2. K fertilizer was applied as a base fertilizer and top dressed at the V6 stage with a ratio of 1:1.

In the ONM pattern, plant density was 82,500 plants ha^–1^ (Planting density recommended for yield enhancement in local trials^30^) and fertilization rates were as follows: 360 kg N ha^−1^, 225 kg P ha^−1^, and 405 kg K fertilizer ha^−1^. All of the P was applied as basal fertilizer for each treatment. N was split into two forms for application: a basal fertilizer and a fertilizer dressed at growth stages V6, V12, and one week after the VT stage with a ratio of 1:2:5:2. K fertilizer was applied twice, at a ratio of 1:1 for both basal and topdressing fertilizer (V6).

In the FP pattern, plant density was 60,000 plants ha^–1^(the common and commercial recommended planting density of local farmers) and fertilization rates were as follows: 322.5 kg N ha^−1^, 112.5 kg P ha^−1^, and 195.0 kg K fertilizer ha^−1^. N was split into two forms for application: basal fertilizer and fertilizer dressed at the V12 stage at a ratio of 1:1. P fertilizer was applied twice, at a ratio of 4.8:5.2 for both basal and topdressing fertilizer (V6). K fertilizer was applied twice, at a ratio of 1:4 for both basal and topdressing fertilizer (V6).

### Agronomic management and measurements

**Figure 1:**
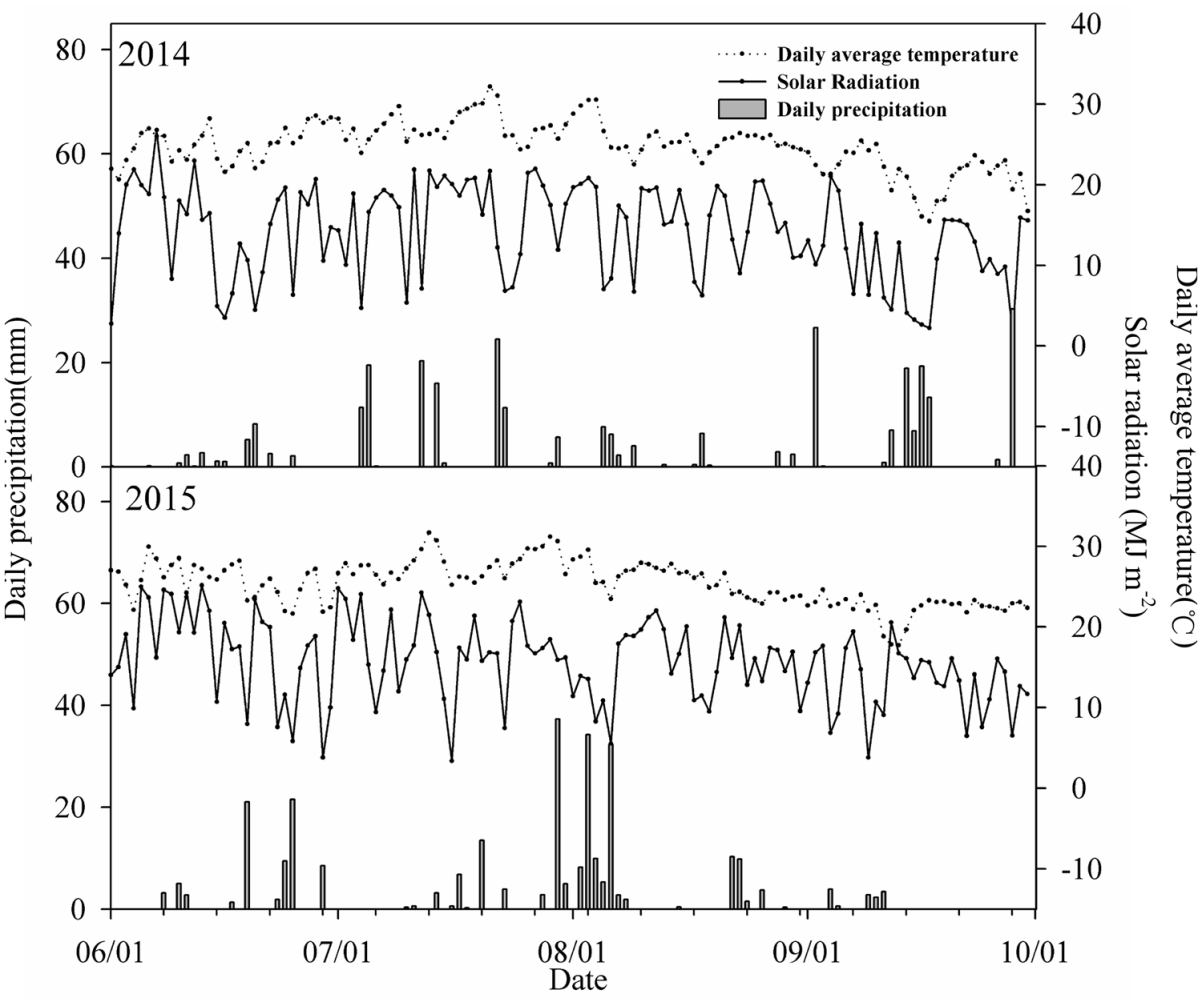
Meteorological data for the summer maize growing seasons in 2014 and 2015.

Field management was conducted by the collaborating farmers, but with guidance from the researchers. Seeds were sown on 15 June and harvested on 1 October in both years. Figure 1 shows the precipitation, solar radiation, and average temperatures for the whole growth period in 2014 and 2015. Pest and weed control were used in line with local high-yield practices. All of the plots were well irrigated.

Soil sampling (at 0–40 cm depth) occurred before maize sowing. Twelve plants were sampled at the R1 and R6 stages for tissue nutrient analysis. Plant samples were divided into four parts: stalk (including stem, leaf sheaths, and the tassel), leaves, husk and cob, and grain. All of the samples were heat-treated at 105 °C for 30 min, dried at 70 °C to a constant weight, weighed to obtain biomass, and then ground into a fine powder (and passed through a 2-mm mesh screen). Plant and grain samples were digested with H_2_SO_4_-H_2_O_2_. Total N and P concentrations were determined using a Bran + Luebbe continuous-flow analyzer (Bran + Luebbe, Hamburg, Germany). Total K was determined using a Sherwood M410 flame photometer (Sherwood M410, Cambridge, England)^34^. For yield and component measurements, 18-m^2^ samples in the center of the plots were harvested and the number of harvested plants was counted. Grain yields were expressed as actual ears per unit area, grain number per ear, and 1,000-grain weights^13^.

### Calculation

We collected plant samples to analyze N, P, and K concentrations. Biomass and nutrient recovery efficiency transport index included the biomass (nutrient) remobilization amount (RA, defined as the difference between the biomass (nutrient) amount at R1 stage and the biomass (nutrient) amount at R6 stage for a vegetative organ), biomass (nutrient) remobilization efficiency (RBE, defined as the ratio of the remobilization amount to biomass (nutrient) amount at R1 stage ), and apparent contribution to grain biomass (nutrient) by biomass (nutrient) remobilization (AC, defined as the ratio of the remobilization amount to grain biomass (nutrient) amount). Equations shyam were used for both specific vegetative organs and the total amount of vegetative organs (leaves, stalks, and husk and cob) to calculate biomass or nutrients. The calculation patterns are as follows^35^:

$$ {\text{RA}}_{i} = S_{i} - M_{i} $$

$$ {\text{RBE}}_{i} = (S_{i} {-}M_{i} )/M_{i} \times 100 $$

$$ {\text{AC}}_{i} = \left( {S_{i} {-}M_{i} } \right)/G_{i} \times 100 $$

where i is the biomass or nutrient (N, P, or K); S is the biomass or nutrient accumulated at the R1 stage (kg ha^−1^); M is the biomass or nutrient accumulated at the R6 stage (kg ha^−1^); and G is the grain biomass or nutrient accumulated at the R6 stage (kg ha^−1^).

The total nutrient uptake, nutrient use efficiency including nutrient recovery efficiency (RE, defined as recovery of nutrients in fertilizer by plant per unit area), harvest index (HI, defined as the ratio of grain nutrient content to whole plant nutrient content), partial factor productivity (PFP, defined as the ratio of grain yield to fertilizer amount under the application of a certain fertilizer), and AE of the nutrient application were calculated as follows^36^:

$$ {\text{RE}}_{i} = \left( {U_{i} {-}U0_{i} } \right)/F_{i} $$

$$ {\text{HI}}_{i} = UG_{i} /U_{i} $$

$$ {\text{PFP}}_{i} = Y/F_{i} $$

$$ {\text{AE}}_{i} = \left( {Y - Y0_{i} } \right)/F_{i} $$

where i is the nutrient (N, P, or K); F is the amount of fertilizer applied (kg ha^−1^); Y is the grain yield (kg ha^−1^); Y0 is the yield (kg ha^−1^) in the control treatment with no N, P, or K; U is the total plant nutrient uptake in above-ground biomass at the R6 stage (kg ha^−1^); UG is the grain nutrient uptake in above-ground biomass at the R6 stage; and U0 is the total plant nutrient uptake in aboveground biomass at the R6 stage in a plot with no N, P, or K (kg ha^−1^).

### Statistical analyses

An ANOVA analysis using SPSS 13.0 software (SPSS Inc., Chicago, IL, USA) was performed on the differences among SH, ONM, and FP at the 0.05 level. A two-way ANOVA analysis using SPSS 13.0 software was performed on the differences of years and cultivation patterns at the 0.05, 0.01, and 0.001 levels. Graphs were produced with Sigma Plot 10.0 software.

## Results

### Yield and yield components

We found significant differences in yield, actual ears, grain number per ear, and 1,000-kernel weight among the three different cultivation patterns (Table 2). In two years, the SH and ONM increased average yield and actual ears by 35.4 and 20.7%, and by 20.2 and 17.6%, respectively, compared with the FP. But SH and ONM reduced average grain numbers per ear by 11.7 and 5.9%, and 1,000-kernel weight by 7.7 and 5.5%, respectively. The reason for the high yield with the SH and ONM is that the number of actual ears increased significantly more than with the FP. However, the actual ears and yield in 2014 were lower than in 2015. This may be because the effective accumulated temperature and the solar radiation in 2015 was higher than in 2014. The cultivation pattern and year interaction were not significant for these parameters.

**Table 2:** Grain yield and its components under different cultivation patterns.

Year	Patterns	Actual ears (× 10^4^ ha^−1^)	Grains number (ear^−1^)	1,000-kernels weight(g)	Yield (kg ha^−1^)
2014	SH	8.67a	536.81c	306.02b	11,859.63a
	ONM	7.67b	542.7b	306.99b	11,446.95b
	FP	6.74c	595.28a	329.07a	9,920.65c
2015	SH	8.94 a	427.18 c	350.24 b	12,419.21 a
	ONM	7.97 b	464.32 b	352.10 b	12,203.19 b
	FP	6.27 c	496.05 a	368.42 a	10,197.64 c
Year (Y)		NS	***	***	***
Cultivation pattern (C)		***	**	***	***
Y × C		NS	NS	NS	NS

SH, super high-yielding cultivation pattern; ONM, optimized nutrient management cultivation pattern; FP, local farmer's practice cultivation pattern. Different small letters indicate significantly differences at *P* < 0.05 as determined by the LSD test. NS, no significant; *, ** and ***, F-values significant at 0.05, 0.01 and 0.001 levels.

### Biomass and nutrient accumulation during pre-silking and post-silking

**Figure 2:**
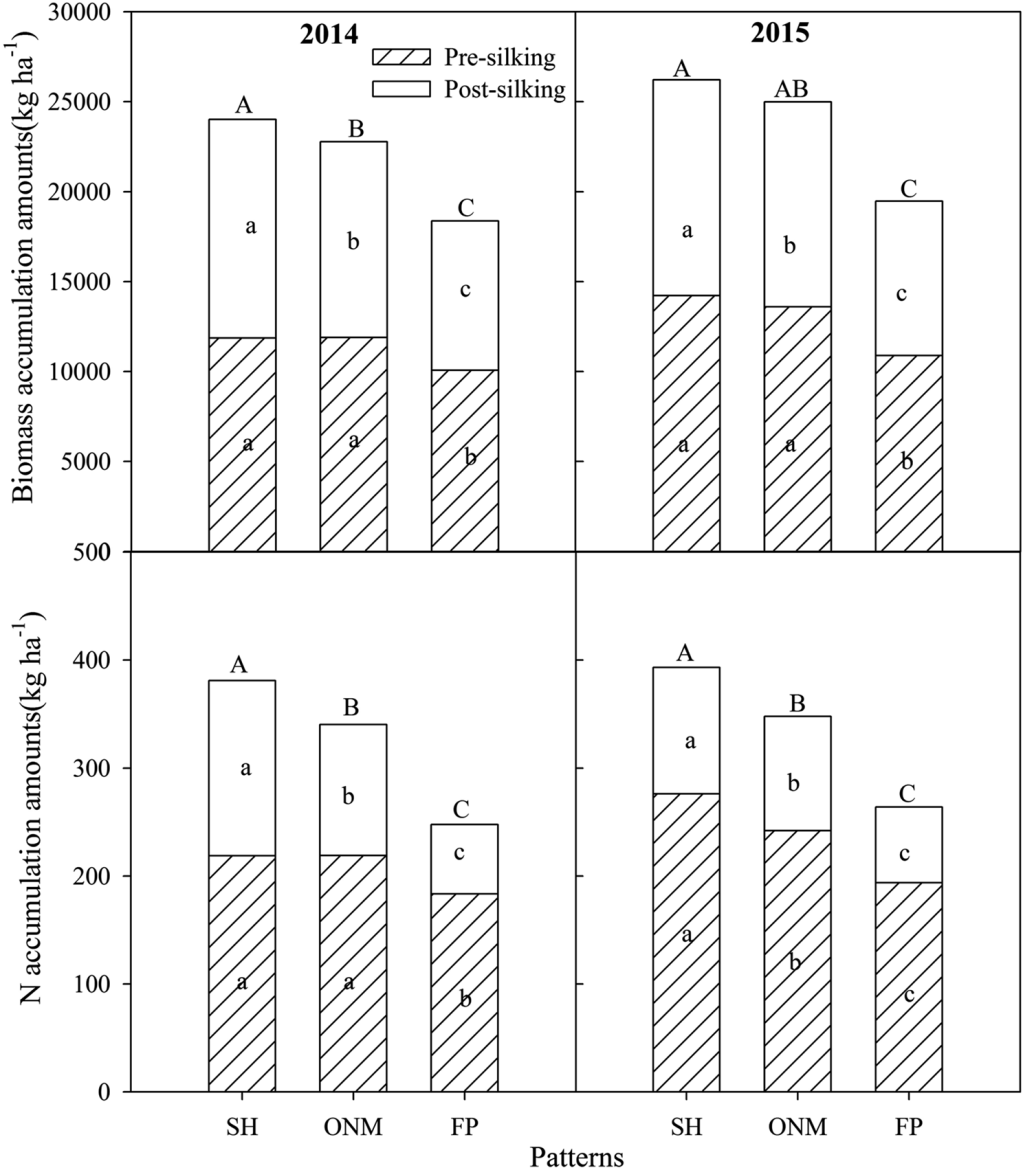
Biomass and N accumulation amounts and ratios in the pre-silking and post-silking stages under different cultivation patterns over two years (n = 12). SH, super high-yielding cultivation pattern; ONM, optimized nutrient management cultivation pattern; FP, local farmer's practice cultivation pattern. Different small letters indicate significant differences among biomass and N accumulation amount under different stage, and different capital letters indicate significant differences among biomass and N accumulation amount under whole growth stages (*P* < 0.05).

**Figure 3:**
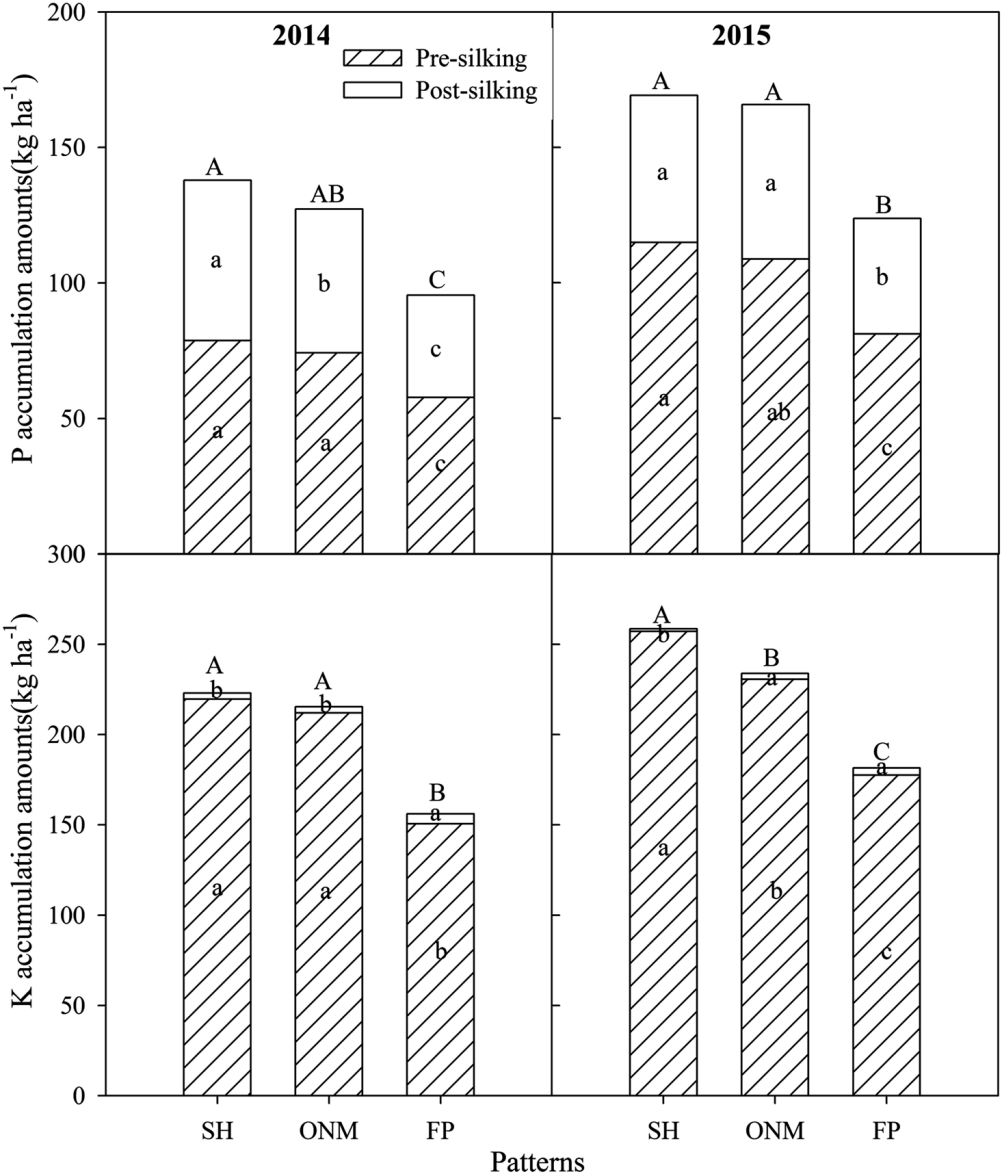
P and K accumulation amounts and ratios in the pre-silking and post-silking stages under different cultivation patterns over two years (n = 12). SH, super high-yielding cultivation pattern; ONM, optimized nutrient management cultivation pattern; FP, local farmer's practice cultivation pattern. Different small letters indicate significant differences among P and K accumulation amount under different stage, and different capital letters indicate significant differences among P and K accumulation amount under whole growth stages (*P* < 0.05).

Significant differences in biomass and nutrient accumulation at the R1 and R6 stages were found among the different cultivation patterns. Compared with FP, the SH and ONM increased biomass, and N, P, and K accumulation amounts at the R1 stage by 24.4, 31.2, 39.4, and 34.8% and by 21.7, 22.2, 31.7, and 34.8%, respectively; SH and ONM increased biomass, N, P, and K accumulation at the R6 stage by 32.7, 51.3, 40.0, and 42.5% and by 26.3, 34.5, 33.7, and 32.9%, respectively (Figs. 2 and 3).

Biomass, N, P, and K accumulation before silking were significantly higher than during post-silking. Across the two years, about 49.4–55.9% biomass, 57.4–74.0% N, 57.1–67.9% P, and 96.5–99.4% K accumulated before silking. The biomass and N accumulation amount during post-silking with SH and ONM were higher than those with FP; P accumulation during post-silking differed between the three cultivation patterns in the two years; most of the K uptake during pre-silking and K uptake with FP during pre-silking were lower than with the other cultivation patterns(Figs. 2 and 3.).

### Distribution of biomass and nutrients at R6 stage

**Figure 4:**
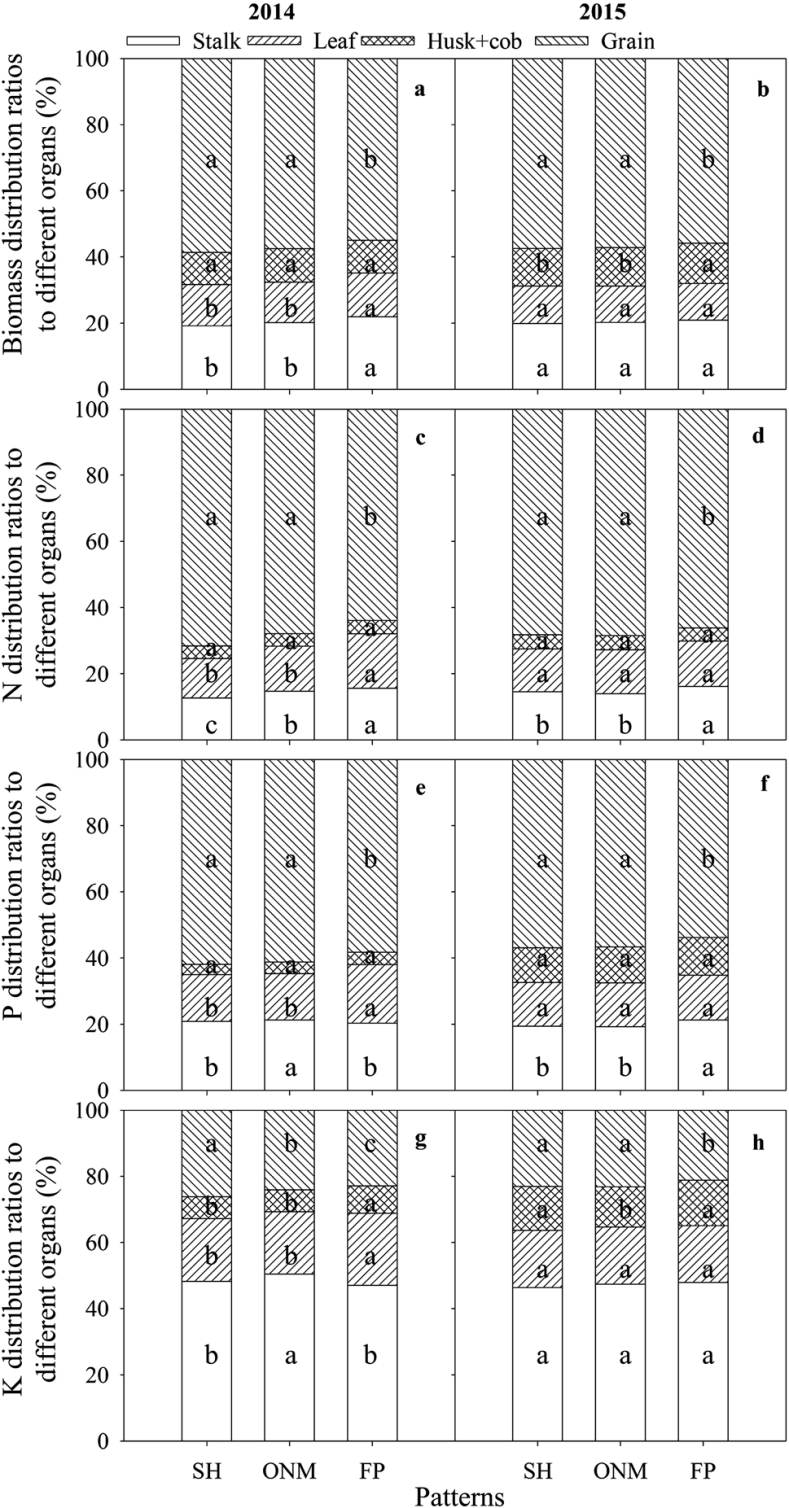
Biomass and nutrient distribution ratios to different organs in the R6 stages under different cultivation patterns over two years(n = 12). SH, super high-yielding cultivation pattern; ONM, optimized nutrient management cultivation pattern; FP, local farmer's practice cultivation pattern. Different small letters indicate significant differences among biomass, N, P, and K distribution ratios to different organs (*P* < 0.05).

In the R6 stage, more biomass, N, and P were present in the grain, and more K was present in the stem. The grain biomass distribution for SH and ONM was significantly higher than for FP in both years. Biomass distribution to the vegetative organs with the SH and ONM was significantly lower than with the FP. Grain N was the same as grain biomass allocation. The N distribution to the vegetative organs (stalk + leaf + husk + cob) was significantly higher under FP than under the other patterns. Stalk P distribution with the SH and ONM was higher than with FP, but leaf P distribution was lower than with FP. Stalk P distribution with SH and ONM was significantly higher than that with FP. Grain K distribution was similar to grain P distribution, and vegetative organ K distribution was higher in the R6 stage with FP than with the other cultivation patterns (Fig. 4).

### Remobilization of biomass and nutrients

The cultivation patterns significantly affected the total RA of biomass (Table 4). Across the two years, the average RA among the three patterns was positive for biomass. The RA values of the biomass from the stalk and the leaf were significantly higher under SH and ONM than under FP, and the RA of the biomass from the husk and the cob was lower under SH and ONM than under FP. Compared with FP, SH and ONM increased the total RA of the biomass by 29.7 and 30.0%, respectively (Table 3). The cultivation patterns significantly affected the total RBE of the biomass. The patterns significantly affected stalk RBE and leaf RBE (Table 4). The biomass total RBE was significantly lower with FP than with the other cultivation patterns in 2015, while no significant difference was observed in 2014 (Table 3). The total apparent contribution of remobilization to the grain was lower with the SH and ONM than with FP. The remobilization contribution to the grain from the stem did not differ between the SH and ONM, and the remobilization contribution to the grain from the leaf was significantly reduced. The cultivation pattern and year interaction were also significant for the total RA, the efficiency, and the apparent contribution of grain to biomass (Table 4).

**Table 3:** Biomass and nutrient remobilization amounts, remobilization efficiencies, and apparent contribution to the grain among different cultivation patterns over 2 years.

Biomass and nutrient	Year	Patterns	Remobilization amount (kg ha^−1^)				Remobilization efficiency (%)				Apparent contribution to grain (%)			
			Stalk	Leaf	Husk + cob	Total	Stalk	Leaf	Husk + cob	Total	Stalk	Leaf	Husk + cob	Total
Biomass	2014	SH	593.5a	865.4a	− 292.7c	1,166.2a	11.4a	22.5b	− 2.1b	10.5a	4.2a	6.1b	− 2.1b	8.2c
		ONM	522.1b	907.3a	− 194.0b	1,235.4a	10.2b	24.4a	− 1.5a	11.3a	4.0a	6.9ab	− 1.5a	9.4b
		FP	436.2c	733.7b	− 146.6a	1,023.3b	9.8b	23.3ab	− 1.5a	11.0a	4.3a	7.3a	− 1.5a	10.1a
	2015	SH	765.8a	791.8a	− 162.5b	1,395.1a	12.8a	21.1a	− 1.1ab	11.1a	5.1a	5.3a	− 1.1ab	9.3a
		ONM	685.2b	795.4a	− 148.2b	1,332.4a	11.9ab	22.4a	− 1.0a	11.1a	4.8a	5.6a	− 1.0a	9.4a
		FP	527.7c	609.8b	− 125.7a	951.8b	11.5b	22.0a	− 1.2b	9.9b	4.9a	5.6a	− 1.2b	9.3a
N	2014	SH	21.5a	68.1a	6.1b	95.7a	31.0a	59.8a	2.2c	47.0a	7.9a	25.0c	2.2c	35.1c
		ONM	18.1b	64.2b	6.8b	89.1a	26.6b	58.1a	3.0b	44.9b	7.8a	27.8b	3.0b	38.6b
		FP	12.0c	55.6c	8.4a	76b	23.6c	57.8a	5.3a	45.9b	7.6a	35.2a	5.3a	48.1a
	2015	SH	31.7a	70.9a	15.3ab	117.9a	35.6a	58.2a	5.7b	48.5a	11.8ab	26.5a	5.7b	44.0b
		ONM	29.9a	57.5b	14.4b	101.8b	38.3a	55.3b	6.0b	48.1ab	12.6a	24.2b	6.0b	42.8c
		FP	20.0b	44.8c	16.2a	81c	32.0b	55.1b	9.3a	47.6b	11.5b	25.6ab	9.3a	46.4a
P	2014	SH	6.0a	10.4a	5.0a	21.4a	17.3a	34.8a	5.9b	28.9a	7.0a	12.2a	5.9c	25.1a
		ONM	3.9b	9.5a	5.0a	18.4b	12.5b	34.6a	6.4b	27.1ab	5.0b	12.2a	6.4b	23.6b
		FP	1.7c	7.2b	4.1b	13c	7.9c	29.8b	7.3a	24.6b	3.0c	12.9a	7.3a	23.2b
	2015	SH	19.0a	8.4a	3.6a	31a	36.7a	27.5a	3.7a	29.8a	19.7a	8.8a	3.7a	32.2a
		ONM	17.9a	7.9a	0.0c	25.8b	35.9a	26.4a	0.0c	26.4b	19.0a	8.4b	0.0c	27.4b
		FP	8.8b	6.0b	1.2b	16c	25.0b	26.3a	1.9b	21.8c	13.2b	9.1a	1.9b	24.2c
K	2014	SH	24.8a	24.7a	0.7a	50.2a	18.7a	36.8a	1.3c	23.3a	42.6a	42.5b	1.3b	86.4a
		ONM	19.4b	21.8b	0.8a	42b	15.2b	34.9a	1.5b	20.4b	37.5b	42.0b	1.5b	81.0b
		FP	8.8c	15.9c	0.7a	25.4c	10.7c	31.8b	2.0a	17.4c	24.6c	44.3a	2.0a	70.9c
	2015	SH	29.0a	25.7a	− 3.6b	51.1a	19.4a	36.6a	− 6.1b	20.4a	48.6a	43.1a	− 6.1b	85.6a
		ONM	25.2b	21.4b	− 3.3b	43.3b	18.5a	34.6b	− 6.1b	19.4a	46.4b	39.5b	− 6.1b	79.8b
		FP	13.5c	15.8c	− 0.1a	29.2c	13.4b	33.5b	− 0.4a	16.9b	35.1c	40.9b	− 0.4a	75.6c

SH, super high-yielding cultivation pattern; ONM, optimized nutrient management cultivation pattern; FP, local farmer's practice cultivation pattern. Different small letters indicate significantly differences at *P* < 0.05 as determined by the LSD test.

**Table 4:** Variance analysis of the remobilization amounts, remobilization efficiencies, and the apparent contributions of the remobilization of biomass and various nutrients.

Biomass and nutrient	Source of variationt	Remobilization amount (kg ha^−1^)				Remobilization efficiency (%)				Apparent contribution to grain (%)			
		Stalk	Leaf	Husk + cob	Total	Stalk	Leaf	Husk + cob	Total	Stalk	Leaf	Husk + cob	Total
Biomass	Year (Y)	***	***	***	*	***	*	***	*	***	***	***	NS
	Cultivation pattern (C)	***	***	***	***	**	NS	***	***	NS	**	***	*
	Y × C	NS	NS	***	**	NS	NS	***	***	NS	NS	***	*
N	Year (Y)	***	**	***	***	***	NS	***	***	***	***	***	**
	Cultivation pattern (C)	***	***	***	***	***	NS	***	***	NS	***	***	***
	Y × C	*	**	NS	*	**	NS	*	NS	NS	***	*	**
P	Year (Y)	***	***	***	***	***	NS	***	NS	***	***	***	***
	Cultivation pattern (C)	***	***	***	***	***	***	***	***	***	NS	***	***
	Y × C	***	NS	***	***	NS	***	***	***	***	NS	***	**
K	Year (Y)	***	NS	***	NS	***	NS	***	***	***	NS	***	NS
	Cultivation pattern (C)	***	***	***	***	***	**	***	***	***	NS	***	***
	Y × C	NS	NS	***	NS	*	NS	***	***	NS	NS	***	NS

SH, super high-yielding cultivation pattern; ONM, optimized nutrient management cultivation pattern; FP, local farmer's practice cultivation pattern. NS, no significant; *, ** and ***, F-values significant at 0.05, 0.01 and 0.001 levels as determined by the LSD test.

The cultivation patterns significantly affected the total RA of N, P, and K (Table 4). The RA values of N and K from the stalk and leaf were significantly higher under the SH and ONM than under FP, and the RA of N from the husk and cob was lower under SH and ONM than under FP. Compared with FP, SH and ONM increased the total RA of N, P, and K by 36.0, 80.7 and 85.5%, and by 21.6, 52.4 and 56.2%, respectively (Table 3). The cultivation patterns significantly affected the total RBE of N, P, and K and the stalk RBE of N, P, and K, but only had a significant effect on the leaf RBE of P and K (Table 4). The total RBE of N was significantly higher with the SH and ONM than with the FP in 2015, while no significant difference was observed in 2014. Across the two years, compared with FP, SH and ONM increased the total RBE of P and K by 26.5 and 27.4% and by 15.3 and 16.0%, respectively. The cultivation pattern and year interaction effects were significantly affected by the total apparent contribution to grain of P and K, but not N (Table 4). The cultivation patterns significantly affected the total apparent contribution of N, P, and K to the grain (Table 4). Across the two years, compared with FP, the N total apparent contribution to the grain under the SH and ONM was reduced by 16.3 and 13.9%, respectively, and the P and K total apparent contributions to the grain were increased by 20.9 and 17.4%, and by 7.6 and 9.8%, respectively (Table 3). The C and year interaction effect significantly affected the total apparent contribution to the grain of biomass, N, and P, but not K (Table 4).

### Nutrient use efficiency

ONM produced considerably higher N use efficiency than SH and FP for both years, with the exception of N HI (Table 5). On average, ONM produced significantly higher N RE, N AE, and N PFP (*P* < 0.05) than SH or FP, with increases of 2.1%, 1.3 kg kg^−1^, and 5.85 kg kg^−1^, and 10.6%, 1.65 kg kg^−1^, and 1.65 kg kg^−1^, respectively. The N HI of the SH was significantly higher than for the ONM and FP, which produced increases of 1.7 and 4.75%, respectively.

**Table 5:** N, P, and K utilization efficiencies of summer maize under different cultivation patterns.

Year	Patterns	RE (%)			HI (%)			PFP (kg kg^−1^)			AE (kg kg^−1^)		
		N	P	K	N	P	K	N	P	K	N	P	K
2014	SH	34.3ab	20.1c	22.9c	71.6a	61.9a	26.1a	27.6c	46.0c	27.6c	6.4b	8.5b	4.2b
	ONM	36.9a	22.5b	26.0b	67.9b	61.2a	24.1b	33.9a	54.2b	30.1b	7.6a	9.7a	5.1a
	FP	24.8c	29.9a	34.5a	63.9c	58.2b	22.9c	31.6b	90.6a	52.3a	6.2c	6.0c	3.8c
2015	SH	35.2ab	17.5c	16.4b	68.2a	56.9a	23.1a	26.4c	43.9c	26.4c	5.8b	6.2c	3.3bc
	ONM	36.8a	21.4b	16.0b	68.5a	56.7a	23.2a	31.8a	50.9b	28.3b	7.2a	7.8a	3.7a
	FP	27.7c	25.6a	22.3a	66.4b	53.7b	21.3b	30.8b	88.2a	50.9a	5.3bc	7.1b	3.4ab

SH, super high-yielding cultivation pattern; ONM, optimized nutrient management cultivation pattern; FP, local farmer's practice cultivation pattern. Different small letters indicate significantly differences at *P* < 0.05 as determined by the LSD test.

The P use efficiency of the FP was significantly higher than for the ONM and SH for both years, with the exception of P HI and P AE (Table 5). On average, the P RE and P AE values with the FP were significantly higher than those of the SH and ONM, showing relative increases of 8.95% and 44.45 kg kg^–1^, and 5.8% and 36.85 kg kg^–1^, respectively. The P HI and P AE values with the FP were significantly lower than those of the SH and ONM, showing relative decreases of 3.45% and 0.8 kg kg^–1^, and 3% and 2.2 kg kg^–1^, respectively.

The K RE and K PFP values were significantly higher under the FP than under the ONM and SH for each year. The K HI and K AE values were significantly lower under the FP than under the other two patterns. On average, the K RE and K PFP values under the FP were significantly higher than under the SH and ONM patterns, showing increases of 8.75% and 24.6 kg kg^–1^, and 7.4% and 22.4 kg kg^–1^, respectively. The K HI and K AE values under the FP were significantly lower than under the SH and ONM, showing decreases of 2.5% and 0.15 kg kg^–1^, and 1.55% and 0.8 kg kg^–1^, respectively.

## Discussion

### Biomass and nutrient accumulation and distribution in different organs among different cultivation patterns

Cultivation management practices can change the nutrient uptake rate in the pre-silking and post-silking stages. Different N rates may have led to the differences in nutrients in the pre-silking and post-silking stages^37^. Pre-silking biomass, N, P, and K accumulation under all three cultivation patterns accounted for 49–55, 57–74, 57–67, and 96–99% of the biomass and nutrient accumulation in all of the stages. Post-silking biomass and N accumulation with the SH and ONM were higher than with the FP, but P and K accumulation showed no significant difference (Figs. 2 and 3). High N application rates can significantly improve the amounts of N accumulation in maize, but will reduce the ratio of pre-silking N accumulation during the whole growth stage^38^. Postponing N application can also be beneficial for post-silking nutrient uptake^39^. Plant density effects on post-silking N can be inconsistent from year to year, but one possible negative impact of higher density on post-silking N is that higher leaf N content and leaf biomass accumulation occur at high density^40^. Compared with the FP, the SH and ONM improved the density and increased the number of fertilizer applications. The SH used two N fertilizer applications, while the ONM used one application after anthesis. This is the reason that N uptake increased in the post-silking stage with SH and ONM and why we obtained high biomass and high grain yield. Most nutrients are present in the stalk and leaf at silking, and more nutrients are transported to the grain at the R6 stage. Biomass and nutrient distribution to the grain under the SH and ONM were higher than under the FP (Fig. 4). Chen et al.^38^ also recorded a high biomass and N distribution ratio in the grain with high density and high N supply.

### Biomass and nutrient remobilization efficiency among different cultivation patterns

In maize, 69–80% of grain N, 18–23% grain P and 44–205% of the grain K was obtained from the remobilization from stalk and leaves^12,41^. And researchers have suggested that high nutritional quality of the grain can be obtained through a combination of post-silking uptake and remobilization from the vegetative organs^20^. Farmers could adjust these two processes by optimizing their cultivation practices^20,37,38^. In our experiment. the total biomass and N apparent contribution to the grain were lower with the SH and ONM than the FP. This means that post-silking N uptake is higher with SH and ONM than with FP (Fig. 4). Mi et al.^42^ noted that stay-green varieties can reach higher yields because they have longer periods of photosynthesis. Higher nutrient remobilization efficiency from the vegetative tissues leads to leaf senescence, such as Wei et al.^43^ showed that stimulating excessively P translocation from the leaf during the period from heading to maturity would hinder photophosphorylation and carbohydrate metabolism, and finally lead to lower grain yield in rice. Lower nutrient remobilization efficiency from the vegetative tissues also decreases yield and lowers nutrient use efficiency because excessive nutrients are stored in the vegetative organs^12,38^. As shown in Table 3, the biomass and nutrient RA and remobilization efficiencies were both significantly higher for SH and ONM than for FP. This is consistent with previous. studies that found that coordination of N remobilization efficiency and N uptake in the post-silking stage was important for high grain yield and high N use efficiency^12^. The SH and ONM enhanced nutrient remobilization efficiency by increasing planting density and increasing the N uptake ratio in the post-silking stage by increasing N application rate after VT.

Venekamp et al.^44^ reported that the redistribution of P from vegetative organs into seeds was restricted under a low N condition. Chen et al.^20^ reported that low N treatment increased N RBE. Ciampitti et al.^40^ reported that all internal efficiencies improved as the N rate increased but decreased as plant density increased. The effects of monoculture management on the remobilization and utilization of maize nutrients have been studied as the above, but the practice of production both adopt a combination of various cultivation measures, and the research on comprehensive cultivation measures is rare, especially the effect on the remobilization of maize. Our experiment shows comprehensive cultivation measures could effectively regulate nutrient RBE. To research the effect of comprehensive cultivation measures on the characteristics of maize remobilization will be the theoretical basis for obtaining higher nutrient utilization efficiency of maize.

### Nutrient use efficiency among different cultivation patterns

Improving the efficiency of nutrient utilization is an effective way to reduce resource waste and decrease the cost of production. Researchers have suggested that reducing the fertilizer rate, adjusting the planting density, and optimizing fertilization are effective ways of improving nutrient use efficiency^1,24,38^. Our cultivation patterns combine these three factors, and we suggest that the ONM has higher N RE, P HI, N PFP, N AE, P AE, and K AE than the other two patterns, and that the other nutrient use efficiency index of the ONM is intermediate between those of the SH and FP. This is because the ONM had an optimized planting density and an optimized N fertilizer application rate and timing compared with the FP and SH. The P PFP and K PFP values were much higher for FP than for SH and ONM. This was because under the P-deficiency treatment the P levels of the soil were too low and the FP had higher soil P content (Table 4). Although P RBE and K RBE were lower than with the other patterns, the P RE and K RE values with FP were higher than with the other patterns because of the low fertilizer use, which was consistent with previous studies. Xu et al.^36^ reported that hybrid maize strategies included site- and season-specific knowledge of crop nutrient requirements and soil indigenous nutrient supplies were required to increase crop yield and nutrient use efficiency with low apparent N loss and low greenhouse gas emissions. Similarly, the ONM can maintain an acceptable yield on the basis of higher fertilizer utilization efficiencies. Although the FP had higher P RE and K RE values, it produced a much lower yield which farmers would not accept. The yield was much higher under the SH than under the other two patterns, but its excessive fertilizer use and lower nutrient use efficiency were not economical.

## Conclusion

As the study of maize cultivation has continued to become more advanced, research on the effects of comprehensive practices on the physiological ecology of maize has become more critical. Reasonable planting density and fertilizer management can effectively regulate the absorption, distribution, remobilization, and utilization of nutrients in maize. In our study, yield increases under the ONM grain averaged 1.82 t ha^–1^ more than the yields of the FP; total RA and total RBE of ONM were intermediate between those of the SH and FP; and RE, PFP, and AE of N, P and K were all higher than those under the SH. We conclude that the ONM is a suitable cultivation pattern for northern China as its increased grain yield, reduced fertilizer input, promoted nutrient distribution to the grain, promoted nutrient RBE, and then increased fertilizer use efficiency.

## Abbreviations

SH: Super high-yielding cultivation
ONM: Optimized nutrient management cultivation
FP: Local farmer's practice cultivation
N: Nitrogen
P: Phosphorus
K: Potassium
RBE: Remobilization efficiency
RA: Remobilization amount
AC: Apparent contribution to grain biomass (nutrient) by biomass (nutrient) remobilization
RE: Recovery efficiency
HI: Harvest index
PFP: Partial factor productivity
AE: Agronomic efficiency
